# Are joint replacement registries cost-effective? Economic evaluation of the Australian orthopaedic association National joint replacement registry

**DOI:** 10.1007/s00402-025-06029-x

**Published:** 2025-08-12

**Authors:** Charles Okafor, Son Nghiem, Carl Holder, Christopher Vertullo, Joshua Byrnes

**Affiliations:** 1 Centre for Health Services Research, University of Queensland, Brisbane, Australia; 2 Department of Health Economics, Wellbeing and Society, Australian National University, Canberra, Australia; 3 South Australian Health and Medical Research Institute, Adelaide, Australia; 4 National Joint Replacement Registry, Australian Orthopaedic Association, South Australia, Australia; 5 Menzies Health Institute, Gold Coast, Australia; 6 Centre for Applied Health Economics, Griffith University, Brisbane, Australia

**Keywords:** Registry, Australia, Cost-utility, Cost-benefit, Benefit

## Abstract

**Introduction:**

There is limited evidence on the cost-effectiveness of joint replacement registries. This study investigates two key questions: (i) Has the Australian Orthopaedic Association National Joint Replacement Registry (AOANJRR) been cost-effective in improving the health outcomes of Australian joint replacement recipients? and (ii) Do the benefits of the registry outweigh its costs?

**Materials and methods:**

A cost-utility and a cost-benefit analysis was performed from the healthcare system perspective, with a secondary analysis from the payer’s perspective. Participants were patients who underwent hip or knee replacements between July 1999 – December 2021. Health outcomes, measured as Quality-Adjusted Life Years (QALYs), revisions averted, and recalled prostheses, were converted to monetary terms using the value of a statistical life year, revision costs and protheses costs. Costs were presented in 2022 Australian dollars, with a discount rate 5% per annum. Decision-making thresholds were set at a willingness-to-pay of AU$50,000/QALY and a benefit-cost ratio of 1.

**Results:**

From the healthcare system perspective, the incremental cost-effectiveness ratio (ICER) was dominant (cheaper and provides better outcomes) (AU$-170,982/QALY), with a benefit-cost ratio of 10.29. From the payer’s perspective, the ICER was also dominant (AU$-60,137/QALY) with a benefit-cost ratio of 10.49. Results remained robust across sensitivity analyses.

**Conclusion:**

The AOANJRR is highly cost-effective, demonstrating significant health and financial benefits. For every dollar spent by the government, approximately nine dollars were saved. Verifying the cost benefits of clinical quality registries is crucial to justify ongoing investments, support informed clinical decisions, and ensure high-quality, accurate data for continuous improvements in patient care and safety.

**Supplementary Information:**

The online version contains supplementary material available at 10.1007/s00402-025-06029-x.

## Introduction

Joint replacement is a major surgical intervention that effectively reduces pain, restores function and improves the quality of life of patients with end-stage joint disease [1]. The Australian Orthopaedic Association (AOA) recognised the need to establish a national joint replacement registry in 1993, commencing initial data collection in 1999 and becoming fully national in mid-2002 for hip and knee replacement [1]. The AOA National Joint Replacement Registry (AOANJRR) was established to enhance the quality of care for joint replacement patients by providing accurate, reliable data to guide clinical practice. Its aim is to create a voluntary, observational registry that supports patient safety and monitors implant performance to identify and address potential issues early [1]. The AOANJRR has a participation rate of 100% from surgeons and hospitals (private and public). The AOANJRR reports on the clinical outcomes of joint replacement in Australia to surgeons, government, hospitals, patients and prostheses manufacturers through a variety of mechanisms including clinical audits, public reports, peer reveiwed publications, education and partnership [1]. The AOANJRR has been funded by the Commonwealth Department of Health since 1999, and in 2009, legislation was passed by the Federal Parliament to enable recovery of the AOANJRR funding from the orthopaedic industry [1].

A compelling example of the value of registry data is the recall of the Articular Surface Replacement (ASR) metal-on-metal hip replacement [1], with the AOANJRR being the first registry in the world to flag the higher than anticipated risk of revision associated with the use of this prosthesis [1]. The ASR was recalled from the Australian market in December 2009 and in December 2010, a worldwide recall on ASR was issued due to the increasing effect of metal toxicity and revisions [1]. On 29 June 2016, the Australian Federal Court approved the settlement of the “DePuy ASR Hip Implant Class Action” for AU$250 million plus interest and legal costs [2] **.**

Although joint replacement registries offer numerous benefits [3], only some of these advantages can be directly measured [4]. When confounders are controlled, the impact of joint replacement registries in identifying and reporting optimal clinical practices and prostheses can be assessed through a variety of measures including the quality of life of the patients who undergo joint replacement, ‘time to first revision’, the burden of revision surgeries after primary joint replacement and recalled prostheses. However, many important benefits, such as improved surgical decision-making and surgeon preferences [5], increased patient confidence, avoided litigation costs, stimulation of prostheses design improvements, and the promotion of a safety-focused clinical culture, remain difficult to quantify.

Assessing the value for money of the AOANJRR is critical for guiding future decisions regarding its operation and funding. However, there is limited evidence on the registry’s cost-effectiveness, particularly when comparing its ongoing annual costs to its broader, often intangible benefits. Prior studies have struggled to isolate the registry’s true economic impact due to overlapping interventions and benefits, a lack of non-registry comparisons, spillover effects (the transfer of scientific knowledge or clinical practices from a registry country to a non-registry country), and global surgical advancements (global trend, e.g. use of robotic-assisted surgery), leading to potential misclassification of both measurable and non-measurable benefits [6].

This study aimed to provide evidence (from the healthcare system and payer’s perspectives) of the value for money of the AOANJRR in promoting recommended clinical practices by conducting a cost-utility analysis and a cost-benefit analysis based on differences in registry and non-registry periods health outcomes, while controlling for global trend and spillover effect on hip and knee replacement. The benefit measures of the registry included in this study were quality-adjusted life-years (QALY), cost saved from averted revisions and cost of recalled prostheses. For the purpose of this study, healthcare system perspective considers all the costs and benefits that directly impact the healthcare system, i.e., costs borne by hospitals, clinics, insurers, and government health agencies, rather than patients or society as a whole. The payers perspective only considers the direct costs to the government (e.g. federal and state). While the healthcare system and payer perspectives provide robust insights into direct medical costs, this analysis excludes broader societal costs (lost productivity, care-giver and rehabilitation costs, patient incurred costs, early retirement, non-revision prosthesis complication management) and long-term economic impacts, due to data limitations, which may underestimate the full value of the AOANJRR.

Descriptions of health economic terminologies used in this study are provided in the Supplementary file (Supplementary file 1).

## Methods

### Setting and sample size

The study was carried out in Australia using the AOANJRR dataset. The sample size used for the study was 87,246 hip revisions and 80,464 knee revisions in Australia from July 1999 to December 2021 [1]. These include index revision and re-revision joint replacements, which could be total or partial revisions (major, intermediate, or minor complexities).

## Intervention and comparator

The intervention in this study is the ‘registry’. A scenario of ‘no registry’ was the base comparator and was compared to the ‘registry’ scenario. The ‘no registry’ scenario was simulated as a ‘global trend and spillover’ scenario. This scenario is the counterfactual of the registry scenario, and it captures the health outcomes that will be observed in Australia regardless of the registry’s impact. For example, Patient Specific Instrumentation (PSI) which was introduced in early 2000s to assist with total knee replacement is a global trend (technological advancement), regardless of the registry’s impact. Likewise, the introduction of robotic assisted surgery is a global trend, which was developed regardless of the registry’s impact. The ‘registry’ scenario was modelled to capture the costs and outcomes associated with the operations of the registry, including the effect of global trend and spillover.

## Model design

The study was performed as a decision analysis. Annual revisions for hip and knee, respectively from July 1999 to December 2021 from the AOANJRR were computed in the model for both scenarios. The input parameters were made probabilistic using the appropriate parametric distributions. The model simulated costs and outcomes for 22 years. Future costs and outcomes in the model were discounted at an annual rate of 5% [7]. Table 1 provides the model parameters and the model assumptions.

## Model input parameters

### Burden of revision

As used in this study, revision burden is the number of revision joint replacements performed in one year divided by the total number of revisions plus primary joint replacement during the same year. Data on the burden of revision for hip and knee from June 1999 to December 2021 were obtained from the AOANJRR [1]. The revision burden data from the AOANJRR represents the burden observed with the effect of the registry, global trend and spillover effect. The revision burden for ‘no registry’ was estimated as the revision burden observed in a counterfactual Australia with no registry, using the registry’s revision burden data [1] and the effectiveness data from a published panel regression model [8] (see Supplemental file 2). A panel regression model combines cross-sectional and time series data, whereby the same cross-sectional unit (e.g. annual revision burden) is measured at different times (e.g. 1999–2021), and the difference in revision burden over the time horizon is analysed.

### Revision mortality and all-cause mortality

Revision mortality rate data were obtained from AOANJRR dataset [1], while the all-cause mortality data were obtained from the Australian Life Table [9].

## Effectiveness of the registry on revision burden

The effectiveness of the registry was measured as ‘changes in revision burden’ based on coefficients from a published panel regression model [8] (also in Supplementary file 2). The regression model used a difference-in-differences method to compare the registry period of registry countries to non-registry periods of registry and non-registry countries, while controlling for confounders ― spillover and global trend effect, which could obscure the effect of the registry on revision burden [10]. The effectiveness of the registry on revision burden was measured as the average revision burden observed with the registry less the effect of global trend and spillover, while the effectiveness of the ‘no registry’ was measured as the average revision burden observed with the effect of global trend and spillover from the study [8]. The effectiveness of the registry was conservatively modelled from the time of full national coverage (July 2002) to account for the registry’s lag effect.

## Health outcome and benefits

The health outcome measure was QALY based on the changes in revision burden (averted revisions) and the health utilities. The health utilities were estimated using the EuroQol five-dimension five-levels (EQ-5D-5L) questionnaire. The EQ-5D-5L questionnaire is a preference-based generic tool that is widely used to measure Health Related Quality of Life (HRQoL) [11]. This generic tool describes and assesses an individuals health state into five dimensions: mobility, self-care, usual activities, pain/discomfort, and anxiety/depression. Each of the five domains is assessed through a five-level scale, with the upper level indicating a higher level of severity (no, slight, moderate, severe, and extreme problems). The health utilities were obtained from the AOANJRR patient-reported outcome measures (PROM) dataset, including pre- and post-revision utilities and averted revision utilities [1]. PROM data collection commenced in September 2017 with 44 hospitals nationally and increased to over 80% in 2022 [1]. Given the similarity of utility values from 2018 to 2021 and the lack of PROM data before 2017, it was assumed the utility scores from 1999 to 2021 were constant. The utility values after primary joint replacement were modelled as the utility values for averted revision. For patients who require revision surgery in the model, a disutility (pre-revision utility) period of six months was assumed, given that the average waiting time for joint replacement in Australia is about 190 days [12]. The QALY was estimated by multiplying the life years by the utility weights.

The revisions averted were converted to monetary benefits (cost savings) based on the cost of hip and knee revision in Australia [13]. Given that about 70% of joint replacement surgeries occur in private hospitals [14, 15], with Medicare accounting for about 11% the surgical cost in the private setting [16], about 38% of the cost savings from averted revision was attributable in the payer’s perspective.

Australia accounts for 2.73% of the global market for hip and knee implants with a market share value of AU$552 million in 2022 and a compound annual growth rate of 11.46% [17, 18]. Between 2012 and 2025, there were 2,554 hip and knee medical devices registered by the Therapeutic Goods Administration (TGA) [19]. The TGA Database of Recalls, Product Alerts and Product Corrections (DRAC) have shown that 70 hip and knee medical devices were recalled between 2012 and 2025 [20], which translates to an average recall rate of 2.67%. In Australia, 20% of all joint prostheses recall can be attributable to the AOANJRR [8]. Using these data, the monetary benefits from prostheses recall was estimated.

The QALY, the monetary benefits from averted revisions and the monetary benefits from recalled prostheses were used to estimate the incremental cost-effectiveness ratio (ICER) and the benefit-cost ratio (BCR). The monetary value of the QALY applied in the BCR analysis followed the Harvard-led approach for cost-benefit analysis [21] and was based on the value of statistical life year (VSLY), using the Australian best practice regulation guidance note on the value of statistical life (VSL) [22], and the 2019 gross national income (GNI) per capita of AU$76,218 [23, 24]. The Harvard-led approach was based on several robust assumptions, including that: (i) the VSLY is constant; (ii) the VSLY is equivalent to the value per QALY or DALY; and (iii) the value per QALY or DALY is constant. Although the assumptions in the Harvard-led approach are not well-supported by theoretical and empirical evidence [25], it is the most feasible, reasonable and applicable approach currently [21]. The VSLY for Australia was AU$213,000 in 2019, while VSLY to GNI per capita multiplier was 2.79 times (i.e., AU$213,000 divided by AU$76,218). Thus, one QALY was valued at 2.79 times the GNI per capita of Australia in 2022.

### The costs

The costs were estimated from the healthcare system perspective. A secondary analysis was also performed from the payer’s (Federal Government) perspective. Costs data for hip and knee revisions were obtained from the Independent Health and Aged Care Pricing Authority (IHACPA), using the 2021 National Efficient Price (NEP) and the ‘Round 24’ average cost weights for hip and knee revisions [13, 26]. The proportion of major and minor hip and knee revisions obtained from the AOANJRR [1] and the cost weights for major and minor hip and knee revisions from IHACPA Round 24 were used to estimate the average cost for hip and knee revisions, but excludes post-acute rehabilitation, outpatient physiotherapy, and long-term recovery expenses. The costs of hip and knee revisions included the direct medical and direct non-medical costs associated with the joint replacement. Data for the costs used to manage the AOANJRR from 1999 to 2021 financial year end (i.e., June 2022) were obtained from the AOANJRR data custodian [1, 27]. The registry funds comprise the Commonwealth government funding and the AOANJRR membership subscription fee [1]. The funds are utilised in data collection, PROM, data storage, validation, analysis, promotion, information dissemination, publications and other related costs related to the operations of the registry. All costs were expressed in 2022 Australian dollars (AU$). Details of the cost parameters are shown in Table 1 and Supplementary file 2.

**Table 1:** Parameters input and distribution in the model

Variable	Mean	Distribution (95% CI)	Source
Mortality rate			
Mortality rate due to revision	0.0145	Beta (0.0084–0.0292)	[1]
All-cause mortality	0.0103	Beta (0.0058–0.0210)	[9]
Effectiveness with the registry			
Hip	0.0104	Log-normal (0.0069–0.0140)	[1, 8]
Knee	0.0115	Log-normal (0.0071–0.0162)	[1, 8]
Effectiveness due to global trend and spillover			
Hip	0.0042	Log-normal (0.0031–0.0052)	[1, 8]
Knee	0.0040	Log-normal (0.0027–0.0054)	[1, 8]
Costs (2022 AU$)			
Cost of hip revision event	52,709	Gamma (39,532–65,886)	[1, 13, 26]
Cost of knee revision event	47,022	Gamma (35,266–58,777)	[1, 13, 26]
Commonwealth government funds to registry (annual)	2,543,476	Gamma (1,907,607–3,179,345)	[1] [AOANJRR data custodian]
Membership subscription (annual)	1,161	Gamma (871–1,451)	[27]
Utility weights			
Utility averted hip revision	0.7891	Beta (0.7702–0.8081)	[1]
Utility before hip revision	0.3453	Beta (0.3218–0.3687)	[1]
Utility after hip revision	0.6362	Beta (0.6245–0.6478)	[1]
Utility averted knee revision	0.7572	Beta (0.7485–0.7659)	[1]
Utility before knee revision	0.4145	Beta (0.4060–0.4229)	[1]
Utility after knee revision	0.6299	Beta (0.6180–0.6418)	[1]
Other parameters			
Proportion of cost savings from averted revision attributed to public hospital (%)	38%	n/a	[14, 15]
Australia global market share of prosthetic devices (%)	2.73%	n/a	[17, 18]
Risk of prostheses recall (%)	2.67%	n/a	[20]
2021 National Efficient Price (AU$)	5597	n/a	[13]
Average hip revision cost weight	9.4174	n/a	[1, 26]
Average knee revision cost weight	8.4012	n/a	[1, 26]
2022 GNI per capita	89,160	n/a	[24]

QHAPDC: Queensland Hospital Non-Admitted Patient Data Collection; QHNAPDC: Queensland Hospital Non-Admitted Patient Data Collection; AOANJRR: Australian Orthopaedic Association National Joint Replacement Registry; SDC: Supplemental digital content; GNI: gross national income; n/a: not applicable

### Data analyses

#### Cost-utility and cost-benefit analyses

The summary of the cost-utility analysis result was expressed as an incremental cost-effectiveness ratio (ICER).

 Where the

$$ \:ICER = \frac{\begin{gathered} (Cost\:of\:operating\:the\:registry \hfill \\ \: - \:benefit\:from\:the\:revisions\:averted \hfill \\ - benefit\:from\:recalled\:protheses) \hfill \\ \end{gathered} }{\begin{gathered} (QALY\:observed\:with\:the\:registry \hfill \\ \: - \:QALY\:observed\:without\:the\:registry) \hfill \\ \end{gathered} }. $$

A willingness-to-pay (WTP) threshold of AU$50,000/QALY was used. Hence, an ICER below the WTP threshold was considered cost-effective. While there is no single agreed WTP threshold value set in Australia, there is evidence to indicate that for pharmaceuticals, new interventions are more likely to be recommended for re-imbursement if they have an ICER below AU$50,000 and is therefore used in this analysis [28].

The summary of the cost-benefit analysis was expressed as a benefit-cost ratio.

Where the

$$BCR = \frac{\begin{gathered} (Monetary\:value\:of\:the\:incremental\:QALY + \: \hfill \\ benefit\:from\:the\:revisions\:averted \hfill \\ + benefit\:from\:recalled\:prostheses) \hfill \\ \end{gathered} }{{Cost\:of\:operating\:the\:registry\:}}. $$

The BCR threshold used was ‘1’, so that a BCR below ‘1’ will be considered not valuable.

One-way sensitivity analysis was performed to assess the extent to which each key variable could affect the results. Probabilistic sensitivity analysis (PSA) was used to assess the simultaneous uncertainty in the model input parameters using a Monte Carlo simulation of 10,000 iterations. All analyses were performed using Microsoft Excel 365.

## Results

From the healthcare system perspective, the discounted total cost to manage the registry from July 1999 to June 2022 was AU$42.19 million, while the discounted total QALY was 53,996. The total QALY for the same period for the ‘no registry’ scenario was 52,465. The monetary benefits due to averted revisions was AU$280.25 million, while the monetary benefits from recalled prostheses was AU$23.75 million. The ICER of the AOANJRR in improving the outcomes of joint replacement in Australia from 1999 until June 2022 was dominant (AU$-170,982/QALY). The benefit from the incremental QALY (AU$130.22 million) in addition to the monetary benefits (costs saved) from averted revision joint replacement and recalled prostheses, yielded a total benefit of AU$434.22 million and a BCR of 10.29. A secondary analysis from the payer’s perspective also yielded a dominant ICER (AU$-60,137/QALY) and a BCR of 10.49.

The Probabilistic Sensitivity Analysis from the healthcare system perspective showed that the registry cost for the period had a 95% CI of AU$49.77 – AU$50.09 million (mean: AU$49.93 million), while the QALY had a 95% CI of 62,996–63,372 (mean: 63,184). The QALY for the ‘no registry’ scenario had a 95% CI of 61,032–61,390 (mean: 61,211). The monetary benefit due to revision joint replacement averted had a 95% CI of AU$363.26 – AU$368.37 million (mean: AU$365.82 million). The 95% CI of the ICER was dominant at lower and upper limits, while the BCR was 10.94–11.39 (mean: 11.16). The Monte Carlo simulation shows that 100% of the iterations were below the willingness-to-pay threshold of AU$50,000/QALY. The one-way sensitivity analysis showed that the major parameters which drive the results include: the effectiveness of the hip registry which could decrease (by 18%) or increase (by 21%) the BCR; the effectiveness of the knee registry which could decrease (by 21%) or increase (by 25%) the BCR; the cost discount rate which could decrease the BCR by 17%; and the outcome discount rate which could increase the BCR by 24%. Despite the uncertainty of these major parameters on the BCR, the one-way sensitivity analysis indicates that changes in the individual parameters’ values did not have a substantial effect on the ICER or BCR to change the policy decision. The multivariate and the one-way sensitivity analyses deemed the results robust (Supplementary file 2). Table 2 shows the results from the PSA, while Table 3 shows the results of the one-way sensitivity analysis.

**Table 2:** Results of the probabilistic sensitivity analysis from the healthcare system perspective

	No registry	Registry
Discounted registry cost (AU$) [CI]	−	49,932,132 [49,773,526–50,090,738]
Discounted QALY [CI]	61,211 [61,032–61,390]	63,184 [62,996–63,372]
Discounted cost saved from revisions averted (AU$) [CI]	−	365,817,507 [363,264,862 – 368,370,151]
Incremental cost (AU$) [CI]	−	-339,637,199 [-342,098,175 – -337,176,224]
Incremental QALY (AU$)	−	1,973 [1,960–1,986]
ICER (AU$/QALY) [CI]	−	Dominant [Dominant – Dominant]
Benefit from incremental QALY (AU$)		167,795,287 [166,710,278–168,880,296]
Benefit from revisions averted (AU$)		365,817,507 [363,264,862 – 368,370,151]
Benefit from prostheses recall (AU$)		23,751,824 [17,813,868–29,689,781]
Total benefit (AU$)		557,364,618 [547,789,009 – 566,940,228]
Net benefit (AU$)		507,432,486 [497,698,271–517,166,702]
Benefit-cost ratio		11.16 [10.94–11.39]

CI: Confidence interval; QALY: Quality-adjusted life-years; ICER: Incremental cost-effectiveness ratio

**Table 3:** One-way sensitivity analysis of the parameters from the healthcare system perspective

Variables	ICER at lower values (AU$/QALY)	ICER at upper value (AU$/QALY)	% change in BCR at lower value	% change in BCR at upper value
Base case	ICER = Dominant		BCR = 10.29	
Revision mortality rate	Dominant	Dominant	− 0.07	+ 0.14
All-cause mortality rate	Dominant	Dominant	+ 0.07	− 0.27
Effectiveness of the registry on hip revision burden	Dominant	Dominant	− 18.46	+ 20.76
Effectiveness of the registry on knee revision burden	Dominant	Dominant	− 20.69	+ 25.22
Cost of knee revision event	Dominant	Dominant	− 5.95	+ 5.88
Cost of hip revision event	Dominant	Dominant	− 5.34	+ 5.27
Annual cost of the registry	Dominant	Dominant	+ 5.48	− 5.00
Pre-revision utility	Dominant	Dominant	+ 0.54	− 0.61
Post-revision utility	Dominant	Dominant	+ 0.41	− 0.47
Averted revision utility	Dominant	Dominant	− 1.01	+ 1.01
Outcome discount rate	Dominant	Dominant	+ 23.53	− 2.70
Cost discount rate	Dominant	Dominant	− 16.57	+ 4.53

ICER: Incremental cost effectiveness ratio; QALY: Quality-adjusted-life years; BCR: Benefit cost ratio

## Discussion

The most important finding of this globally novel cost-utility and conservative cost-benefit analyses of the AOANJRR is that from a healthcare system perspective, the Incremental Cost-Effectiveness Ratio of the AOANJRR was dominant (AU$-170,982/QALY) and the Benefit-Cost Ratio was about ten times the cost of managing the registry, providing a net benefit of about AU$392 million dollars as at June 2022. From the payer’s perspective, the ICER was also dominant (AU$-60,137/QALY) and for every dollar spent by the government on the registry, there was a return on investment of about nine dollars (BCR = 10.49).

The Benefit-Cost Ratio would likely be higher if other potential outcomes which are not feasible to measure were included in the analysis. The Probabilistic Sensitivity Analysis showed that 100% of the iterations were below the Willingness-To-Pay, which implies that the probability of the AOANJRR being cost-effective at the pre-specified WTP is 1, suggesting that there is a 100% likelihood that the AOANJRR is cost-effective. The one-way sensitivity analysis further showed that the results were robust, as changes in the parameter values did not affect the results in a way that could otherwise change or affect policies in response to the results.

Verification of the cost-utility and cost-benefit of clinical quality registries such as the AOANJRR is vital for several reasons. Firstly, it justifies ongoing funding by demonstrating the registry’s value in improving joint replacement outcomes [3]. The AOANJRR requires significant investment in data collection, validation, and reporting, all of which are essential to maintaining a high-quality dataset. A strong benefit-cost ratio supports continued investment, ensuring the integrity of the registry’s data and maximizing its impact on patient care.

Secondly, it facilitates clinical improvement. High-quality data allows the AOANJRR to identify trends, risk factors, and complications associated with joint replacements, enabling surgeons and healthcare providers to make informed, evidence-based decisions. This promotes patient safety, optimizes surgical practices, and reduces revision rates, reinforcing the importance of accurate and comprehensive data collection.

Lastly, it supports research and innovation. Verifying the economic value of the AOANJRR encourages wider research initiatives, improvements in prosthesis design, and advancements in joint replacement techniques. The registry’s data also contribute to global knowledge transfer, influencing best practices in orthopaedics worldwide. By demonstrating its cost-effectiveness, the AOANJRR helps secure ongoing investment, ensuring its role in driving continuous improvements in patient outcomes and healthcare efficiency.

The benefit-cost ratio (10:1, from the payer’s perspective) of this study is comparable to the result of a recent cost-benefit analysis of the registry in Australia [6], when judged from a different analytical scenario. The actual BCR estimate reported by the study [6] was 5:1, but the study demonstrated that the BCR can be up to 10:1–46:1 when supplementary analyses were considered and when compared with international benchmarks. A plausible reason for the difference in BCR between the study [6], and our study was the spillover and global trend effects and recalled prostheses, which were not controlled for in the referenced study.

### Limitations

This study has several limitations, the majority of which could result in underestimating the registry’s benefits. First, it primarily uses annual revision burden to measure the registry’s effectiveness. While valuable, this excludes other potential benefits, such as Enhanced Recovery After Surgery (ERAS), revision rate, quality of life after primary joint replacement, surgeon education, shifts in clinical decision-making, increased patient confidence, reduced litigation costs, avoided patient suffering, improved prosthesis design, and a stronger safety culture, potentially underestimating the registry’s impact [4]. Second, although the AOANJRR collects data on shoulder, ankle, and other joint replacements, limited data for these lower-volume procedures locally and globally restricted analysis to hip and knee replacements. Third, the study excludes broader societal costs, such as out-of-pocket patient expenses, lost productivity, caregiver burden, and rehabilitation costs, which may result in a conservative estimate of the registry’s overall value. Fourth, non-revision poor outcomes, such as patients experiencing pain or implant complications without undergoing revision, are not captured, further underestimating the registry’s benefits. Fifth, as an observational study, causality cannot be established, and the findings may be influenced by unobservable external factors, despite controlling for confounders. Also, it is possibly that the lag effect effect of the registry benefit could potentially span beyond the three years applied in the model. Sixth, data completeness and quality depend on accurate reporting, and the lack of systematic patient-reported outcomes measures limits a full assessment of patient benefit. Lastly, the study assumes that VSLY is equivalent to QALY, which may affect the BCR estimate, though this is a standard approach in economic evaluations [21].

## Conclusion

This study demonstrates that the AOANJRR is highly cost-effective, delivering significant economic and clinical value through reduced revision burden, improved patient outcomes, and informed clinical decision-making. Even with conservative estimations, our findings confirm that for every dollar invested by the government, there is a substantial return on investment, validating the importance of continued funding for the registry. Additionally, the AOANJRR drives improvements in surgical practices, facilitates prostheses recall, and contributes to global knowledge transfer.

Moreover, it is important to recognize that if less easily measured benefits were included in the analysis, the benefit-cost ratio (BCR) would be considerably higher. Broader impacts, such as reduced patient harm, lower litigation costs, and changes in surgeon behaviour, further amplify the registry’s value. These outcomes, though not fully quantified in the model, emphasize that the presented estimates are conservative and likely understate the true impact of the AOANJRR.

Despite some limitations, such as the exclusion of societal costs and non-revision outcomes, this analysis highlights the registry’s essential role in advancing patient safety, promoting evidence-based care, and delivering substantial economic benefits. The AOANJRR stands as a model of how clinical quality registries can improve healthcare outcomes and drive value-based care globally.

## Supplementary Information

Below is the link to the electronic supplementary material.

Supplementary Material 1

Supplementary Material 2

## Data Availability

Data is provided within the manuscript or supplementary information files.
