# Supplementary material for: Are joint replacement registries cost-effective? Economic evaluation of the Australian orthopaedic association National joint replacement registry
Source: Arch Orthop Trauma Surg. 2025 Aug 12;145(1):408. doi: 10.1007/s00402-025-06029-x (PMC12343730; doi:10.1007/s00402-025-06029-x)
Supplement: Supplementary file 1 — Supplementary Material 1 [file 402_2025_6029_MOESM1_ESM.docx]

**Description of Some Health Economics Terms Used in the Study**

1. Spillover effect:

This is an indirect effect where outcomes in a treatment group will impact the non-treatment group. This can occur through knowledge transfer, scientific publications, data sharing etc. When spillover effect is not controlled, it can confound outcomes in a treatment group.

1. Global trend effect:

This refer to technological progress or global advancements which occurs naturally. For instance, fifty years ago televisions can only display black and white images. Today we have smart televisions displaying multiple colours with better contrast and colour separation. The use of robotics in surgery is another global trend. This trend occurs regardless of the registry impact, such that a treatment group and control group will both benefit from a global trend effect. Thus, global trend can also confound outcomes in a treatment group if it is not controlled in a model.

1. Quality-adjusted life-years (QALY):

This is an outcome measure which captures a patient overall wellbeing after adjusting for the impact of a disease on the patient health. A sick person will have a lower QALY compared to a healthy person.

1. Probabilistic variables:

These are variables whose values change simultaneously in a model. Most of the estimates used in the model have uncertainty (upper and lower values; or 95% confidence interval values). Making a variable value probabilistic in model will help to account or simulate for any uncertainty or changes that may occur with the value of that variable over time if it were a real-life scenario.

1. Parametric distribution:

Different measurements have different mathematical functions that best represents their uncertainty values. These mathematical functions are referred to as ‘distribution’. For example, the uncertainty values of a cost (a variable) estimate are best represented using a gamma distribution, while the ‘effectiveness’ of a treatment is best modelled using a log-normal distribution.

1. Discounting:

The value of $100 today is lesser in year 2050. In other words, what $100 can purchase today, it cannot purchase the same thing in year 2050 due to interest rate. Therefore, in order to present future cost (e.g., year 2050 cost) as year 2025 cost, the cost has to be discounted to the present-day value. In this study we discounted at a rate of 5% based on the available evidence in Australia.

1. Panel regression model

This type of model involves the combination of cross-sectional and time series data to measure difference in outcome between two or more groups. Example of a panel regression data is shown below:

| Group | Time series | Cross sectional |  |  |
| --- | --- | --- | --- | --- |
|  | year | Annual score | Treatment period | Notation |
| Group 1 | 2001 | 40 | Before | A |
| Group 1 | 2002 | 55 | Before | A |
| Group 1 | 2003 | 30 | Before | A |
| Group 1 | 2004 | 35 | After | B |
| Group 1 | 2005 | 48 | After | B |
| Group 1 | 2006 | 45 | After | B |
| Group 2 | 2001 | 41 | Before | C |
| Group 2 | 2002 | 59 | Before | C |
| Group 2 | 2003 | 43 | Before | C |
| Group 2 | 2004 | 22 | After | D |
| Group 2 | 2005 | 33 | After | D |
| Group 2 | 2006 | 39 | After | D |

1. Difference-in-differences

This is an analytical approach use to measure causal effect of an intervention.

With reference to item number 7 above, a simple difference-in-differences estimate, assuming Group 2 is the intervention group, will be:

[(D-C)-(B-A)]

1. Incremental cost-effectiveness ratio (ICER):

This is a ratio of the additional cost of an intervention (relative to the comparator) to the additional benefit on the intervention (relative to the comparator).

1. Dominant ICER

A dominant ICER implies that an intervention is not only cost-saving but also more effective than the comparator, meaning it is both cheaper and provides better outcomes than the comparator.

1. Value of statistical life years (VSLY):

The value of risk reduction (e.g., mortality or knee revision) can be expressed as the value of statistical life (VSL) or VSLY. The VSL concept does not represent the value the health economist, an individual or the government places on saving a life. Rather, it represents individuals’ willingness to exchange money for a small change in their own risk (e.g., 1 in 1,000 decrease in the risk of knee revision or risk of dying).

1. Payer perspective

This is an approach or perspective of economic evaluation where the cost considered in the evaluation includes the cost incurred by the payer (e.g., Medicare, and private insurance).

1. Healthcare system perspective

This is an approach or perspective of economic evaluation where the cost considered in the evaluation includes cost incurred within the health system. It is a combination of the healthcare provider (e.g. a hospital system cost) and the healthcare payer (e.g., Medicare) costing.
